# In this month’s *Bulletin*

**DOI:** 10.2471/BLT.14.001114

**Published:** 2014-11-01

In the editorial section, Alistair Woodward (774) reviews progress on climate change and health. Pat Oungpasuk (775) argues that all countries in the Association of South-East Asian Nations should adopt universal health coverage to ensure future economic growth. 

In a focus on WHO guidelines, Jim Mann tells Fiona Fleck (780–781) how WHO surveyed the evidence for new recommendations on sugar intake. Priya Shetty (778–779) reports on recent efforts to make WHO recommendations on HIV treatment more responsive to the realities facing programme managers.

## China

## How long does a vaccine last?

Terence T Lao et al. (782–789) track hepatitis B prevalence in previously-vaccinated pregnant women in Hong Kong SAR.

## Europe

## Human cost of asbestos

Takashi Kameda et al. (790–797) study asbestos-related diseases in the context of national policies on its use.

## Malawi

## A mixed picture

Michael Abouyannis et al. (798–806) find a low prevalence of multidrug-resistant tuberculosis. 

## India

## Every last case

Krycia Cowling et al. (817–825) make the case for better tuberculosis estimates.

United Kingdom of Great Britain & Northern Ireland

Reaching far-flung islands

Esther L Hamblion et al. (836–843) study compliance by overseas territories with the International Health Regulations.

## Solomon Islands

## Responding to natural disasters

Augustine Bilve et al. (844–848) describe the set-up of an early response network.

## Global

## Are hospitals the best source?

Rasika Rampatige et al. (807–816) present a method for reviewing the accuracy of cause-of-death statistics.

## Tracing donors and recipients

Sarah L White et al. (826–835) discuss globalization of organ transplants.

